# Teledermatology in times of COVID‐19

**DOI:** 10.1111/1346-8138.15812

**Published:** 2021-03-14

**Authors:** Pia‐Charlotte Stadler, Sonja Senner, Surina Frey, Benjamin M. Clanner‐Engelshofen, Leonie H. Frommherz, Lars E. French, Markus Reinholz

**Affiliations:** ^1^ Department of Dermatology and Allergy University Hospital LMU Munich Munich Germany; ^2^ Dr Phillip Frost Department of Dermatology and Cutaneous Surgery Miller School of Medicine University of Miami Coral Gables Florida USA

**Keywords:** barriers, coronavirus disease 2019 pandemic, eHealth, teledermatology, telemedicine

## Abstract

Remote consultations are likely to grow in importance in the following years, especially if the coronavirus disease 2019 (COVID‐19) pandemic continues. Patients’ opinions on teledermatology have already been analyzed, but a current analysis during the COVID‐19 pandemic is lacking. The purpose of this survey was to investigate the satisfaction of patients who had received dermatological advice via telephone during the COVID‐19 pandemic and to analyze their general opinion about eHealth as well as possible limitations for a broad implementation. Ninety‐one patients managed in the dermatology department using telephone consultation during the COVID‐19 pandemic were interviewed. An anonymous questionnaire, including the established quality of life questionnaire (Dermatology Life Quality Index [DLQI]), was used. It was found that men were more satisfied with telephone consultations than women (*p* = 0.029), educational level and age did not correlate with satisfaction (*p* = 0.186 and 388, respectively), and the longer the waiting time for a telephone consultation, the lower the satisfaction (*p* = 0.001). Grouped analysis of all participants showed that the majority (54.0% n = 38/71) were “very happy” with the telephone consultation. Higher disease burden (DLQI) was associated with lower satisfaction (*p* = 0.042). The main stated reasons for using telemedicine were shorter waiting times (51.6% n = 47/91) and no travel requirement (57.1% n = 47/91). Almost one‐quarter (23.1% n = 21/89) of patients would use teledermatology in the future, 17.6% (n = 16/89) would not, and 57.1% (n = 51/89) would only use it in addition to a traditional consultation with personal contact. In conclusion, most patients in the study group still preferred traditional face‐to‐face medical consultations to telephone consultations, but also desired an add‐on telemedical tool. Dermatological care using more modern telemedicine technologies than telephone conferencing is needed to better address patients’ desires, especially in times of the COVID‐19 pandemic.

**Abbreviations**
COVID‐19: coronavirus disease 2019
DLQI: Dermatology Life Quality Index

## 1 INTRODUCTION

In the last decade, eHealth services have evolved and become more relevant for clinical health‐care systems over the world. In 2015, the German eHealth act was adopted and thus integrated technologies, such as electronic health insurance cards, electronic patient records, medication summary, and video consultations in the German legislation.^1^ Nevertheless, to date only 50% of German hospitals have electronic patient records and only 13% provide electronic discharge letters for outside practitioners via portal or mail. Electronic communication with patients is used by 19% of German doctors in private practice and is thereby slightly more developed. Overall, Germany lags behind Austria and Switzerland in eHealth development, which may be due to missing incentives, an eHealth law focusing on the outpatient sector rather than on the inpatient sector, and very stringent data protection laws.^2^ However, as the technological progress and the evolution of mobile phones proceeds rapidly, the perceived utility of telemedicine is now increasing and eHealth is becoming increasingly relevant for daily clinical business.^3^ Teledermatology was one of the first telecommunication technologies to be implemented in 1995 and significantly grew in the last decade.^4^ One can distinguish between two types of teledermatology services: the store‐and‐forward method or the live‐interaction method. The store‐and‐forward method is more common as it is easily manageable concerning time issues and practicable across different time zones. In contrast, the live‐interaction method requires a significant bandwidth, especially for video consultations, but may save time through direct interaction.^5^ The diagnostic and management decisions are reliable and accurate in both methods, and clinical outcomes are similar to those of standard care.^6^, ^7^, ^8^, ^9^, ^10^, ^11^ The main barriers for a broad implementation of eHealth and telemedicine were summarized by Kruse *et al*.^12^ in 2018 and included barriers for organizations, such as costs, reimbursement, legal liability, as well as barriers for patients, such as age and level of education, as well as barriers for staff and programmers such as technical challenges and a resistance to change. In this eHealth survey, we analyzed the opinion and satisfaction of patients consulted via telephone for skin problems during the coronavirus disease 2019 (COVID‐19) pandemic.

## 2 METHODS

After ethical approval by the local ethics commission was obtained, the survey was sent to 340 patients, who had used telephone consultations of the Department of Dermatology and Allergy of the Ludwig‐Maximilian‐University in Munich^13^ during the COVID‐19 pandemic. At that time, we had a complete lockdown and outpatient care was only possible for critically ill patients, so the patients had to use telemedicine to consult the dermatology department. The survey was sent to the patients after a time period of approximately 4 weeks after the phone call. Ninety‐one out of 340 patients completed the questionnaire. Participants were questioned concerning the reason for the consultation, their daily use of digital media, and their satisfaction with the telecommunication.

Data were analyzed using SPSS Statistics version 26.0 (2019; IBM, Armonk, NY, USA) and correlations between different variables were measured by using the Mann–Whitney *U*‐test, Kruskal–Wallis test and χ^2^‐test. The results were interpreted as statistically significant when *p* ≤ 0.05.

The Dermatology Life Quality Index (DLQI) measures the limitation of the quality of life of people with skin diseases during the previous 7 days. The points are added together, with a range of 0–30 points, where 0 indicates no impact and 30 represents a very severe impairment of their quality of life.

## 3 RESULTS

### 3.1 Demographics

**FIGURE 1:**
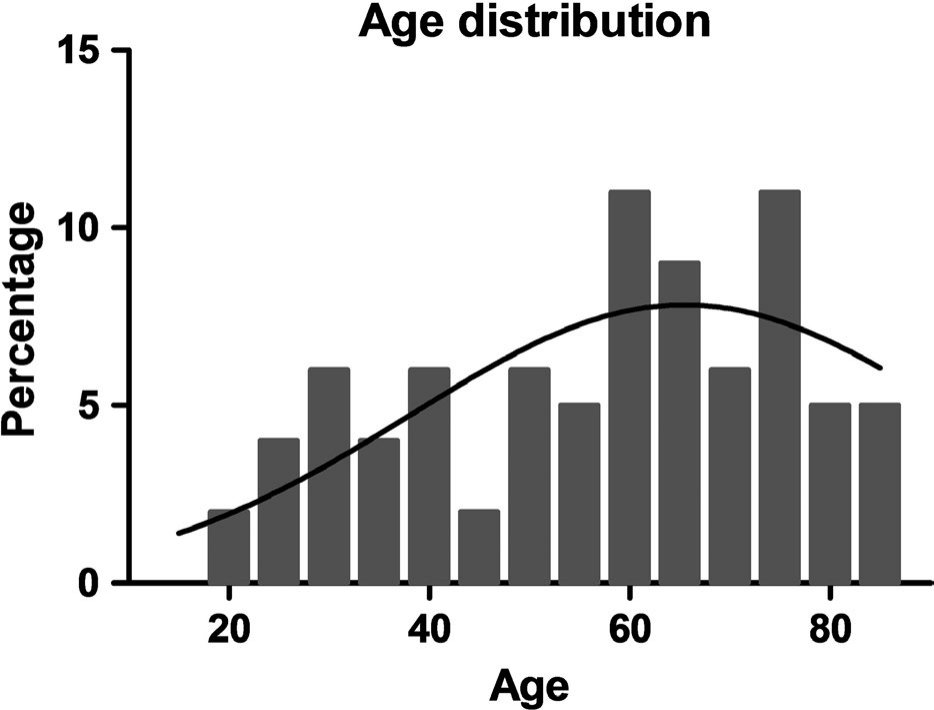
Age distribution in our patient collective with a mean of 55.9 years (standard deviation, 19.2), n = 82

A total of 91 patients took part in our survey (Table 1). The participants were between 18 and 84 years of age, with a mean of 55.9 years (Figure 1).

**TABLE 1:** Overview of the characteristics of our patient collective

	n = 91	
	N	%
Sex		
Female	33	37.4
Male	50	53.8
Intersexual	0	0.0
No gender information	9	8.8
Social status		
Married	45	49.5
Single	18	19.8
Divorced	9	9.9
Widowed	10	11.0
No information	9	9.9
Age (years)		
18–29	8	8.8
30–49	20	25.0
50–59	12	15.0
60–84	42	51.2
No information	9	9.9
School graduation		
University degree	19	20.9
High‐school graduation	17	18.7
Others	53	60.4
No information	12	13.2
Health insurance		
Statutory	82	91.1
Private	1	1.1
No information	8	8.8

### 3.2 Reasons for telephone consultation and subjective factors

**FIGURE 2:**
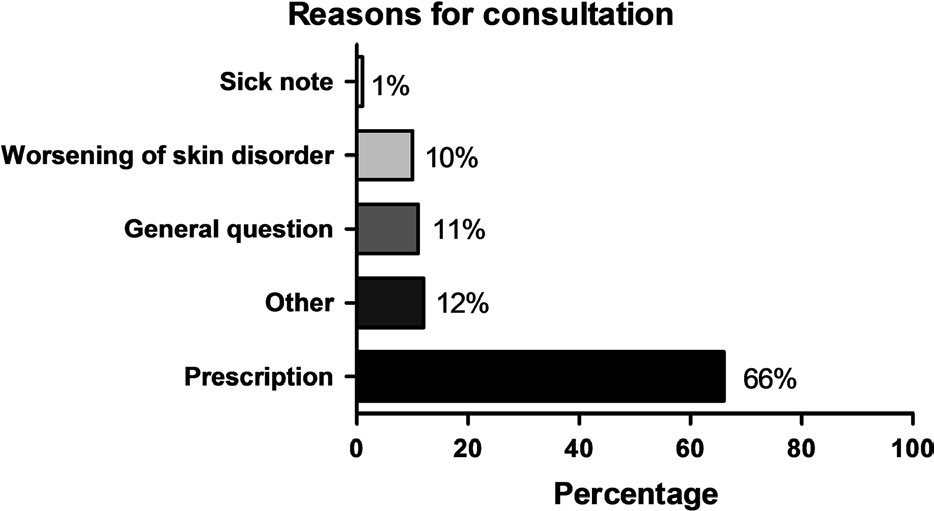
Reasons for consulting a doctor by telephone in times of coronavirus disease 2019: prescription (65.9%), other (18.0%), general question (11.0%), worsening of skin disorder (11.0%), and sick note (1.0%), n = 76

The patients stated the following reasons for the telephone consultation (Figure 2): two‐thirds (65.9%, n = 76) took advantage of the telephone consultation for renewing prescriptions; only one patient wanted a documented sick leave; 1.0% (n = 76) of respondents reported a worsening of their skin condition; and 11.0% (n = 76) had questions.

The clear majority of patients (82.4%, n = 91) who took advantage of a telephone consultation during the COVID‐19 pandemic suffered from chronic (i.e. years) skin disease; for example, autoimmune diseases (20%, n = 91), atopic dermatitis or hand eczema (19%, n = 91), allergic diseases (7%, n = 91), psoriasis (7%, n = 91), acne or rosacea (4%, n = 91), sexually transmitted diseases (3%, n = 91), or others (46%, n = 91). Only one patient had an acute skin disease and 3.0% (n = 91) had a disease existing for months.

**FIGURE 3:**
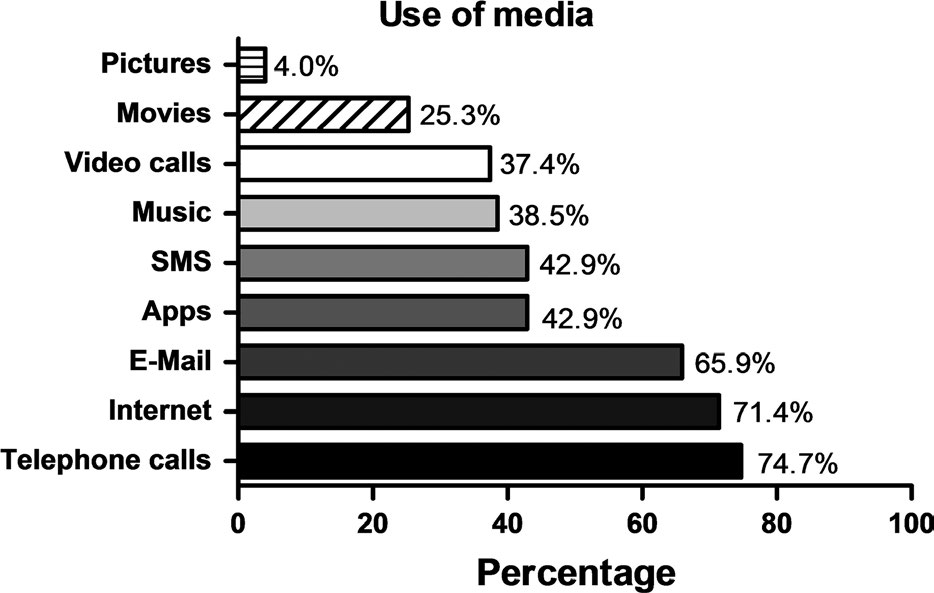
Use of media in times of coronavirus disease 2019: telephone calls (74.7%), internet (71.4%), email (65.9%), applications (42.9%), SMS (42.9%), music (38.5%), video calls (37.4%), movies (25.3%), and pictures (4.0%), n = 82

The waiting time for the telephone consultation was 1 day for 30.0% (n = 91) of the patients. Of these, 17.6% (n = 91) had to wait 2–3 days, 11.0% (n = 91) 3–7 days, 11.0% (n = 91) over 1 week, and 7.7% (n = 91) several weeks, while 23.1% (n = 91) did not provide any information. The clear majority of those surveyed (86.8%, n = 91) also used digital media in their leisure time, and only 12.1% (n = 91) did not. The patients mainly used smartphones (76.9%, n = 91) and laptops (64.8%, n = 91), but also tablets (34.1%, n = 91), e‐book readers (14.3%, n = 91), and other devices (4.4%, n = 91). Figure 3 shows what the media were used for (Figure 3).

One‐third of patients surveyed used digital media 1–2 h a day, 19.8% (n = 91) for more than 30 min a day, 17.6% (n = 91) for less than 30 min, 11.0% (n = 91) between 2 and 4 h, and only 4.4% (n = 91) used digital media more than 4 h a day.

Regarding the DLQI of participants, the minimum was 0 and maximum 27 points. The mean was 6.19 and median 4.00 (standard deviation, 6.540).

### 3.3 Satisfaction

In our survey, men were more satisfied with the telephone consultation than women (*p* = 0.029). Education level and age did not correlate with satisfaction (*p* = 0.186 and 0.388, respectively). The longer the waiting time for a telephone consultation, the lower the satisfaction (*p* = 0.001). The higher the disease burden (DLQI) the lower the satisfaction with the telephone consultation (*p* = 0.042).

**FIGURE 4:**
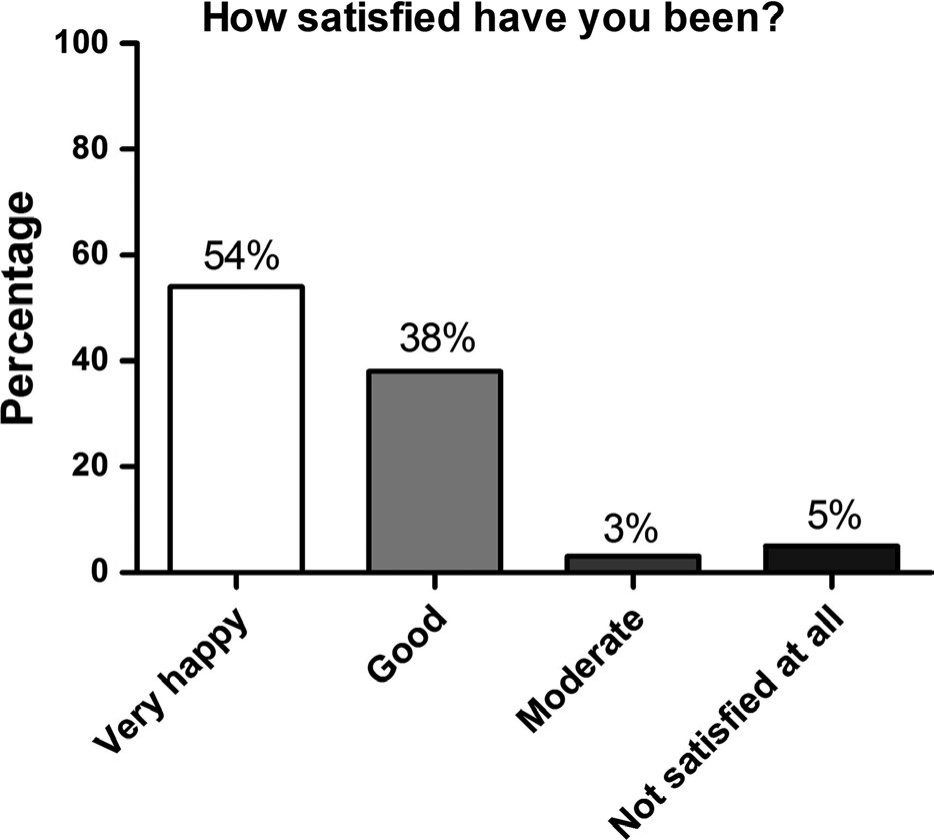
Patient satisfaction with the telephone consultation: “very happy” (54.0%), “good” (38.0%), “moderate” (3.0%), and “not satisfied at all” (5.0%), n = 71

Of all participants, 54.0% (n = 71) were “very happy” with the telephone consultation, 38.0% (n = 71) felt it was “good”, 3.0% (n = 71) “moderate”, and 5.0% (n = 71) were “not satisfied at all” (Figure 4).

Whenever the help was judged as sufficient, the satisfaction was higher (*p* < 0.001).

### 3.4 Telemedicine in the future

The higher the level of education as well as the amount of free time spent with digital media, the more telemedicine was perceived as a useful future option (*p* = 0.001). On the other hand, age and sex did not correlate with the perceived usefulness of telemedicine (*p* = 0.200).

**FIGURE 5:**
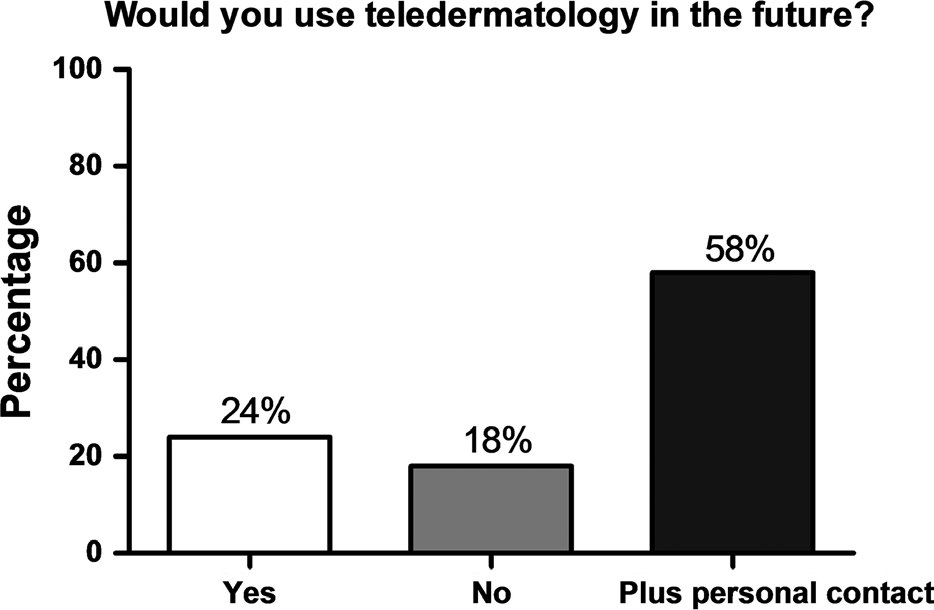
Opinion of the respondents whether they would use teledermatology in the future: yes (24.0%), no (18.0%), and only plus personal contact (58.0%), n = 89

Of the patients surveyed, 24.2% (n = 91) found the idea of having an online medical consultation in the future “very good”, 18.7% (n = 91) “good”, 17.6% (n = 91) “moderate”, 16.5% (n = 91) “not good”, and 17.6% (n = 91) “not good at all”. Regarding use of digital patient care in the future, 23.1% (n = 89) would, 17.6% (n = 89) would not, and 57.1% (n = 89) would only use it in addition to personal contact (Figure 5).

Those who wanted to use digital patient care preferred telephone and video consultation. The main reason for using telemedicine was the expectation of shorter waiting times (51.6%, n = 91) and no travel needs (57.1%, n = 91). Only 8.8% (n = 91) of patients had used other digital medical services since the COVID‐19 pandemic.

The study population would use the digital patient care mostly for follow‐up prescriptions (74.7%, n = 91) or follow‐up consultations (72.6%, n = 91). They also would like to use it for “second opinions” (44.7%, n = 91), sick notes (41.8%, n = 91), or first consultations (28.6%, n = 91). A minority of patients participating in this survey indicated that they would like to use digital patient care for diagnostic issues (17.6%, n = 91).

## 4 DISCUSSION

The present study demonstrates that telephone contact as a mode of telemedicine in times of the COVID‐19 pandemic satisfies a majority of dermatology patients, predominantly due to shorter waiting times and the absence of travel time or expenses. In the literature, the definition of satisfaction is not consistent across surveys and therefore difficult to compare.^14^ Moreover, only one study in the last 10 years exclusively addressed patient satisfaction related to live‐interaction teledermatology.^15^ This study showed that teledermatology clinic consultations required less travel time, shorter waiting time, and lower costs, concordant with the results of our survey showing that one‐third of the collective received a response in only 1 day and those were statistically more satisfied with the telephone consultation. Nevertheless, as we have no comparison group using traditional face‐to‐face medical consultations – and therefore the patients who cannot or do not want to use teledermatology are not sufficiently represented – the results should be interpreted carefully. Nonetheless, a high percentage of patients regarded telephone contact during the current pandemic as helpful. Moreover, the clinical outcome measures of eHealth treatment are similar to those of standard care.^7^, ^8^, ^9^, ^10^, ^11^, ^12^

In our patient collective, one‐third could be sufficiently helped with the telephone consultation and nearly half of the patients were “very happy” with the telephone consultation. In contrast to the live‐interaction method of teledermatology, most studies conducted in recent have exclusively evaluated the store‐and‐forward method. This could be because the store‐and‐forward method is more flexible, cost‐efficient, and therefore more attractive.^16^ Nearly 90% of the patients used digital media, especially smartphones, in their daily business, but mostly for calls, and only 40% of them for video calls. Therefore, video consultation may be a problem for those patients. Nevertheless, even an older patient collective is capable and willing to use digital media as in our patient collective with a median age of 57 years. Furthermore, there is a gap between the use of digital media in general and the use of digital media for medical problems, as only 8.8% of the study group used digital media for medical problems in the COVID‐19 pandemic at the time of our survey. Interestingly, men were significantly more satisfied with the telephone consultation compared with women. In addition, we could confirm the findings of Kruse *et al*.^12^ in 2018, showing that education level correlates with a positive opinion regarding the sense of eHealth. This was not the case for age, as age did not significantly correlate with the reported usefulness of eHealth in our survey, conflicting with the results of Kruse *et al*.^12^ in 2018. The patients who want digital media in the medical care system in the future seem to prefer telephone and video consultation according to our survey results and they prefer to use telemedicine only as an add‐on and prefer in‐person examination. Our results are congruent with a controlled study by Marchell *et al*.^17^ from 2017, where patients referred for dermatology consultations were examined in person, by video, and by store‐and‐forward methods. In person examinations were preferred by both patients and dermatologists. In addition, our data revealed that the higher the burden of disease (DLQI), the lower the satisfaction with the telephone consultation. Therefore, patients with a strong subjective burden of disease may profit from in person examination. This is in accordance with results from a previously published study with 70% of patients experiencing clinical improvement after teledermatology visits.^10^ However, we performed a cross‐sectional study and not a prospective study, which has the disadvantage of not knowing how quality of life changes over time. Another limitation could be that we sent the survey to the patients via postal mail, thus potentially favoring responses from a relatively old patient collective with a median of 57 years and might have led to a very low response rate of around 26%. For younger patients, an online tool might have positively affected the response rate. Furthermore, the majority of our patients suffered from chronic skin disease, as we predominantly treated already known patients. Another reason could be that those patients, who were suffering from a chronic disease, may be more open‐minded for telephone consultations, as their main desire was a new prescription and not a diagnostic issue. This reflects the statement of the responders as to how and for what they would use digital media in the future: the majority would use it for follow‐up consultations and follow‐up visits, so it would not be used by a majority for acute problems.

In conclusion, medical management using telephone consultations may be beneficial for prescription issues, as well as time‐related and spatial barriers. The results underline the importance that telephone consultations should be further integrated in the German legislation to diminish barriers for reimbursement and legal liability. The option of digital treatment is a promising future alternative, at least as an additional consultation option for patients with chronic diseases. The present study shows that teledermatology using telephone consultations is a useful add‐on consultation method in times of the COVID‐19 pandemic, but further research is needed to determine the long‐term satisfaction, feasibility, and effectiveness of real‐time teledermatology.

## CONFLICT OF INTEREST

None declared.
